# Euendolithic Cyanobacteria and Proteobacteria Together Contribute to Trigger Bioerosion in Aquatic Environments

**DOI:** 10.3389/fmicb.2022.938359

**Published:** 2022-07-06

**Authors:** Guimei Wu, Aiyou Huang, Yanhong Wen, Hongxia Wang, Jiangxin Wang, Fuguang Luo, Mingcan Wu

**Affiliations:** ^1^State Key Laboratory of Marine Resource Utilization in South China Sea, College of Oceanology, Hainan University, Haikou, China; ^2^Liuzhou Aquaculture Technology Extending Station, Liuzhou, China; ^3^Institute of Hydrobiology, Chinese Academy of Sciences, Wuhan, China; ^4^College of Life Sciences and Oceanography, Shenzhen University, Shenzhen, China

**Keywords:** carbonate bioerosion, euendoliths, aquatic environment, Cyanobacteria, Proteobacteria, carbon sequestration

## Abstract

Shellfish, mussels, snails, and other aquatic animals, which assimilate limestone (calcium carbonate, CaCO_3_) to build shells and skeletons, are effective carbon sinks that help mitigate the greenhouse effect. However, bioerosion, the dissolution of calcium carbonate and the release of carbon dioxide, hinders carbon sequestration process. The bioerosion of aquatic environments remains to be elucidated. In this study, the bioerosion of *Bellamya* spp. shells from the aquatic environment was taken as the research object. *In situ* microbial community structure analysis of the bioerosion shell from different geographical locations, laboratory-level infected culture, and validated experiments were conducted by coupling traditional observation and 16S rRNA sequencing analysis method. Results showed that bioeroders can implant into the CaCO_3_ layer of the snail shell, resulting in the formation of many small holes in the shell, which reduced the shell’s density and made the shell fragile. Results also showed that bioeroders were distributed in two major phyla, namely, Cyanobacteria and Proteobacteria. Cluster analysis showed that *Cyanobacteria* sp. and two unidentified genera (Burkholderiaceae and *Raistonia*) were the key bioeroders. Moreover, results suggested that the interaction of Cyanobacteria and other bacteria promoted the biological function of “shell bioerosion.” This study identified the causes of “shell bioerosion” in aquatic environments and provided some theoretical basis for preventing and controlling it in the aquatic industry. Results also provided new insights of cyanobacterial bioerosion of shells and microalgae carbon sequestration.

## Introduction

Burning fossil fuels and increasing concentration of greenhouse gases [carbon dioxide (CO_2_)] in the atmosphere intensify global warming, leading to a series of environmental problems. A recent United Nation Climate Change Conference of the Parties (December 2019) held in Madrid updated the climate action plans to at least keep global warming under the 1.5°C limit (Intergovernmental Panel on Climate Change, 2018). Hence, carbon capture and sequestration technology has become one of the prioritized research topics worldwide. Among others, some mollusks construct their shells with limestone [calcium carbonate (CaCO_3_)] by absorbing both calcium ions (Ca^2+^) and bicarbonate (HCO_3_^–^) under the complex cellular and physicochemical processes, leading to carbon sequestration (Wilbur et al., 1983; Tang et al., 2011; Zhang et al., 2012; Arcenegui-Troya et al., 2020; Alonso et al., 2021). However, some microorganisms caused the CaCO_3_ sequestered in the shell to dissolve into the water through bioerosion, causing the CaCO_3_ to become CO_2_, which was released back into the atmosphere (Tribollet et al., 2006). It has been reported that these microorganisms can decompose CaCO_3_ and release CO_2;_ equivalent to 20% of CO_2_ emitted from human activities (Tribollet et al., 2006). Therefore, microorganisms that erode CaCO_3_ of mollusk shells have a significant impact on carbon sequestration and the balance of CO_2_ in the atmosphere.

The aquatic organisms involved in bioerosion include microorganisms (e.g., flagellated fungi, bacteria, and microalgae) and externally visible sponges (Glynn and Manzello, 2015). Cyanobacteria and green algae involved in bioerosion have been widely studied in marine or salt lake areas (Garcia-Pichel et al., 2010; Prusina et al., 2015; Iha et al., 2021; Ndhlovu et al., 2021), especially some euendolithic Cyanobacteria of the morphogenera *Hyella*, *Solentia*, *Plectonema*, and *Mastigocoleus* living in the ocean (Garcia-Pichel et al., 2010). After bioerosion, many tiny holes left in the shell can be observed by scanning electron microscopy (SEM) (Tribollet et al., 2006; Pawłowska et al., 2008). As the main component of these shells was CaCO_3_, according to the formula CaCO_3_ (s) + H^+^↔Ca^2+^ + HCO_3_^–^, some scholars believe that microorganisms can erode shells by secreting acidic substances, causing the shells to produce Ca^2+^ and HCO_3_^–^, and finally the CO_2_ produced was released into the atmosphere (Schneider and Campion-Alsumard, 1999). However, Garcia-Pichel et al. (2010) rejected the proposed mechanism. They suggested that Cyanobacteria were able to take up Ca^2+^ at the excavation front, decreasing the local extracellular ion activity product of calcium carbonate enough to promote spontaneous dissolution there. Intracellular Ca^2+^ was then transported away along the multicellular cyanobacterial trichomes and excreted at the distal borehole opening into the external medium. In addition, Iha et al. (2021) found that some bacteria provide vitamin B_12_ to the marine green alga *Ostreobium* during bioerosion of coral skeletons. Meanwhile, the *Ostreobium* provide photoassimilates (e.g., polysaccharides) to these bacteria, while corals provide nutrients (e.g., ammonium ions) to the bacteria and *Ostreobium*, establishing stable interaction among themselves. Therefore, the participation of other bacteria in the bioerosion process has a non-negligible role.

Microbial populations are highly complex, with up to 80% of them—referred to as the “microbial dark matter” –unculturable (Rashid and Stingl, 2015). However, Sanger sequencing analysis of biomarker genes like stocktickerRNA polymerase beta subunit (*rpoB*), 16S ribosomal stocktickerRNA (16S rRNA), and others (*nifD, recA*, *gyrB*, and *fusA*) helped overcome the dependence on culture method and enabled the discovery of many previously unknown microorganisms (Holmes et al., 2004). Notably, 16S is ubiquitous in prokaryotes and has the characteristics of highly conserved sequence regions. It can be used to design specific or universal primers. It is the most widely used single target for phylogenetic studies of different species of archaea and bacteria (Langille et al., 2013; Ortiz-Estrada et al., 2019; Oyewusi et al., 2020). Therefore, gene analysis methods have excellent advantages for analyzing microbial population dynamics and predicting functions at both *in situ* ecology and laboratory levels. However, gene analysis methods have rarely been applied to analyze microbe-mediated bioerosion of shells.

In recent years, the scale of artificial breeding of shellfish and snails has been growing (Gu et al., 2020), which supports both economic growth and ecological benefits. However, snail shells have often bio-eroded in the constructed aquatic environment, resulting in the death of the snails, and the release of the sequestered carbon into the atmosphere, increasing the greenhouse effect. Bioerosion has only been reported under natural conditions in oceans and saltwater lakes, but a similar effect in artificially created aquatic environments has rarely been investigated.

In this study, we collected snails from aquatic environments representing different geographical locations, observed the microscopic characteristics of bio-eroded shells, and then used 16S rRNA amplification and sequencing technology to identify two parts of *in situ* microorganisms and the laboratory-level infected microorganisms responsible for the shell’s bioerosion. Additionally, target microorganisms were screened for bioerosion verification. This study will (i) elucidate the potential microbes responsible for shell bioerosion in aquatic environments, (ii) fill the research gaps of microbial bioerosion in the aquatic ecosystems, and (iii) provide new insights into reducing carbon emissions and increasing carbon sequestration.

## Materials and Methods

### Collection of Snail Shells

**FIGURE 1:**
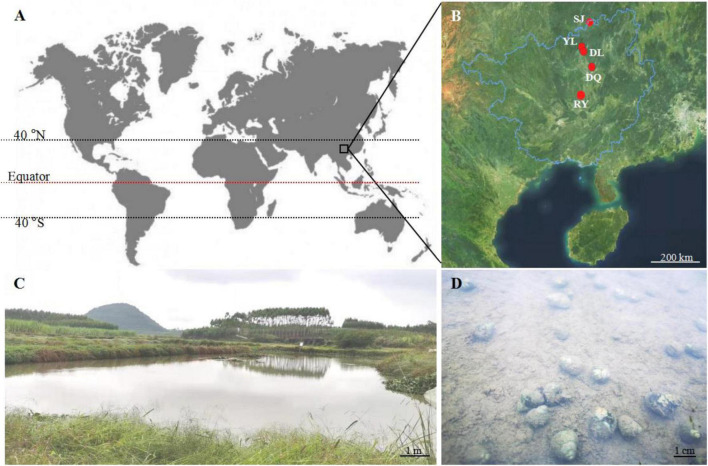
Geographical location of *Bellamya* spp. snail shell collection. **(A)** The location of the collection site on the world map; **(B)** the geographical location of the five aquatic areas where snail shells were collected; **(C)** the aquatic environment of YL; **(D)** the eroded snail in the aquatic water of YL; SL, Sanjiang County (25°56′36″ N, 109°28′41″ E); YL, Yongle Town (25°2′7″ N, 109°8′4″ E). DL, Dongliang Village (25°0′54″ N, 109°13′29″ E); DQ, Dongquan Town (24°32′30″ N, 109° 33′13″ E); RY, Renyi Village (23°43′56″ N, 108°55′32″ E).

*Bellamya* spp. snails were obtained from five different aquatic environments in the Guangxi Zhuang Autonomous Region, China, near the tropic of cancer (Figures 1A,B). From high latitude to low latitude, the aquatic environments included Sanjiang County (SJ, 25°56′36″ N, 109°28′41″ E), Yongle Town (YL, 25°2′7″ N, 109°8′4″ E) (Figures 1C,D), Dongliang Village (DL, 25°0′54″ N, 109°13′29″ E), Dongquan Town (DQ, 24°32′30″ N, 109° 33′13″ E), and Renyi Village (RY, 23°43′56″ N, 108°55′32″ E). The severely bio-eroded snail shells were used as the experimental group, and the healthy shells were used as the control group (CG) (Figure 1D).

### Experimental Design

For *in situ* experiments in the wild, 30 snails were collected from each aquatic environment. The sludge on the shell of the snail was cleaned using sterile water. All biological tissues were removed, leaving only the shell. The hammer-crushed shells were transferred to a centrifuge tube containing 1 ml of sterile water, and then sent to Wekemo Tech Group Co., Ltd., (Shenzhen, China) for 16S rRNA amplification, sequencing, and analysis. A part of the snail shells was fixed with formalin to make electron microscope slices for SEM observation.

For the laboratory-level infected culture experiment, nine tanks (length × width × height = 120 cm × 78 cm × 78 cm) were set up in three groups, containing three tanks in each group. The severely bio-eroded snails collected in the wild aquatic environment were mixed healthy snails and designated as an artificial infection group (AIT). Only severely bio-eroded snails were named as a natural infection group (NI). The feeding conditions in the laboratory were 25°C, light 50 μmol m^–2^s^–1^, unstirred water, and feeding *Chlorella* every day. After 3 months, the morphological changes in snail shells were observed and the biological tissues were removed, the shells were crushed, and sent to Wekemo Tech Group Co., Ltd., (Shenzhen, China) for 16S rRNA amplification, sequencing, and analysis. The liquid from the NI experimental group was coated on solid BG11 plates (Rippka et al., 1979) containing the antibiotic ampicillin (100 mg/L) to screen for potential microalgae with bioerosion function (named CSB03). CSB03 was transferred to a triangular flask with a light of 50 μmol m^–2^s^–1^, 30°C, and a working solution of 200 ml for culture and morphological identification. Finally, all bio-eroded shells were transferred to 4°C for 7 days in the dark and then collected the pigments penetrated from the shells.

For the bioerosion verification experiment, 30 g of the eroded shells from the NI group were transferred to a 500-ml triangular flask containing 200 ml of BG11 medium under the unstirred water. BS-D, a control experimental group, was set up at 30°C and under no light conditions. A BS-L experimental group was also constructed under similar cultural conditions as BS-D except the light conditions, which were at 100 μmol m^–2^s^–1^. Based on the culture conditions of the BS-L, CSB03 algal cells (OD_750_ = 0.3) were inoculated into the medium to create a BS-CSB03-L experimental group. Another experimental group, the S-CSB03-L, was established under similar conditions to BS-CSB03-L except the bio-eroded shells, which were highly sterilized. The calcium ion (Ca^2+^) concentration in the medium from different experiment was measured every other day.

### Experimental Methods

#### Analysis of 16S rRNA Sequencing Data

The off-machine data obtained by Illumina NovaSeq sequencing platforms were graftedand quality-controlled to pick up clean tags. Then, chimera filtering was conducted to receive adequate data that could be used for posterior analysis. To determine the species composition in each sample, operational taxonomic units (OTUs) were clustered with 97% identity, and all different sequences of OTUs were annotated. For *in situ* microbial population analysis, the top 10 with the most abundance were analyzed at the phylum level and relative abundance histograms and cluster heatmaps were created. For laboratory microbial population analysis, the sample data were analyzed using the principal component analysis (PCA) (Wu et al., 2021). The microbial communities within the individual sample and group were analyzed at the phylum level. Based on the database annotation results, each group was analyzed to generate a functional cluster map. In addition, microbial population analyses were performed using cluster analysis at the genus level, and potential bioeroders were identified.

#### Ultrastructure of Bio-Eroded Shells

The methods of sample preparation and observation using SEM were modified according to Ghisleni et al. (2009). Briefly, for sample preparation, the hammer-crushed shells of the CG and NI were washed, respectively, then selected the three flat and suitable size fragments for making electron microscope slices. Paraffin was melted in an oven at 65°C. The selected shell fragments into the paraffins were to be solidified orderly. After the shell paraffin block was completely solidified, the shell paraffins were inserted into an appropriate size (0.5 cm × 0.5 cm) by a scalpel. Paraffin block with shell fragments was fixed on the sample preparation table with conductive adhesive, and its surface was polished by spraying gold for 2 min. Finally, the samples were observed using a field emission SEM (S-4800, Hitachi, Japan).

#### Ca^2+^ Concentrations

Measurement of Ca^2+^ concentrations was performed using inductively coupled plasma-optical emission spectroscopy (Optima 7000 DV, PerkinElmer, United States) following the method developed by Trevelin et al. (2016).

### Statistical Analysis

Samples were achieved in triplicate, and the average and standard deviation were calculated and presented in the final results. The differences between the control and experimental groups were compared by repeated-measures one- or two-way analysis of variance (ANOVA), followed by pairwise comparisons with Sidak’s multiple-comparisons test (Burnett et al., 2020). All statistical analyses were performed using GraphPad Prism version 9.3 (GraphPad Software, San Diego, CA, United States).

## Results and Discussion

### Microbial Bioerosion of Shells in Aquatic Environments

Bioerosion has been reported to occur frequently in coastal areas (Chacón et al., 2006; Garcia-Pichel et al., 2010; Prusina et al., 2015; Iha et al., 2021). However, there is limited information on inland bioerosion, especially in the aquatic environment. This study found microbial bioerosion of snail shells across five aquatic environments (Figure 1), especially in unstirred water (Figures 1C,D). These results suggest that the bioerosion process widely exists in aquatic environments, regardless of geographical location. It has been reported that bacteria, fungi, and microalgae cause bioerosion of shells (Chacón et al., 2006; White and Booth, 2014; Glynn and Manzello, 2015; Gleason et al., 2017), and this study reported some key bioeroders that may play a significant role in the bioerosion of CaCO_3_ shells in the aquatic environment. We suggest that bioerosion in aquatic environments is likely to have broader economic and environmental consequences as it likely reduces shellfish and snail production and increases CO_2_ emission, respectively.

### Ultrastructure of Bio-Eroded Shells

**FIGURE 2:**
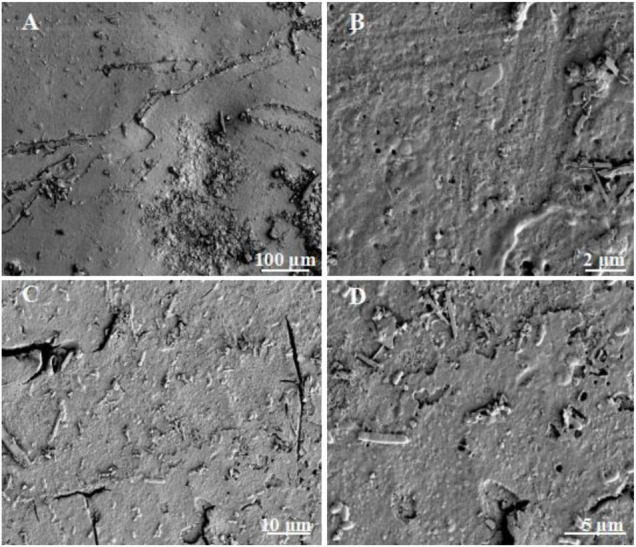
Surface ultrastructure of bio-eroded snail shells. **(A)** The surface of the healthy snail shell; **(B)** the small voids on the surface of the healthy shell; **(C)** the surface of the bio-eroded snail shell, with relatively large cracks; **(D)** the surface of the bio-eroded snail shell, with some microorganisms attached, with some small gaps.

**FIGURE 3:**
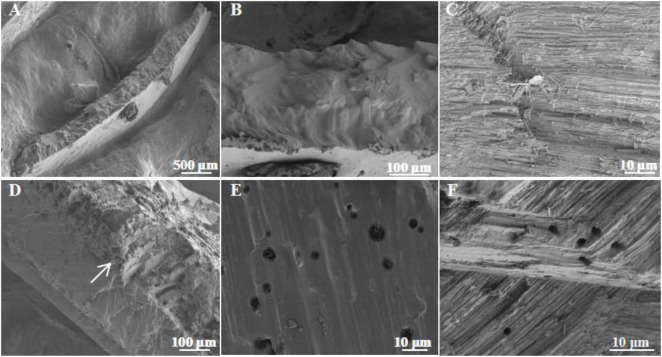
Ultrastructure of the cross section in bio-eroded snail shells. **(A)** The cross section of the healthy snail shell; **(B,C)** the ultrastructure of the cross section of the healthy snail shell; **(D)** the cross section of the bio-eroded snail shell; **(E,F)** the ultrastructure of the cross section in the bio-eroded snail shell, with many small pores with low void density.

Results from this study showed that the healthy shell surface was relatively smooth and clean with a few small voids in between (Figures 2A,B). In contrast, the bio-eroded shell surface was rough and uneven, with more cracks and larger circular voids. Some rod-shaped microbes were attached (Figures 2C,D), suggesting that cracks and voids may be caused by microbial bioerosion, or they may be the “gateway” for microbes to enter the shell for bioerosion. The cross section of a shell showed that the healthy shell was smooth and composed of dense inorganic matter (i.e., CaCO_3_) (Figures 3A–C). However, the bio-eroded shell had a layer of low density and loose calcium material on its surface (Figure 3D). There were many horizontally oriented pores on the shell surface (Figures 3E,F), suggesting that potential bioeroders enter from the shell surface into the shell interior and erode a shell horizontally, which changes the ultrastructure of the shell, reduces the hardness, and increases the fragility. These results are comparable to the findings of Pantazidou et al. (2006), who reported the erosion of estuarine mollusks shells inhabiting saline water lagoons by Cyanobacteria and the formation of similar surfaces and voids inside the shells. However, newly discovered voids on the shell surface (Figures 2C,D) and a relatively loose shell (Figure 3D) in this study implied that the bioerosion characteristics in the aquatic environment were different across ecosystems and need to be further investigated to understand the bioerosion phenomenon.

### *In situ* Microbial Community of the Bio-Eroded Shell

**FIGURE 4:**
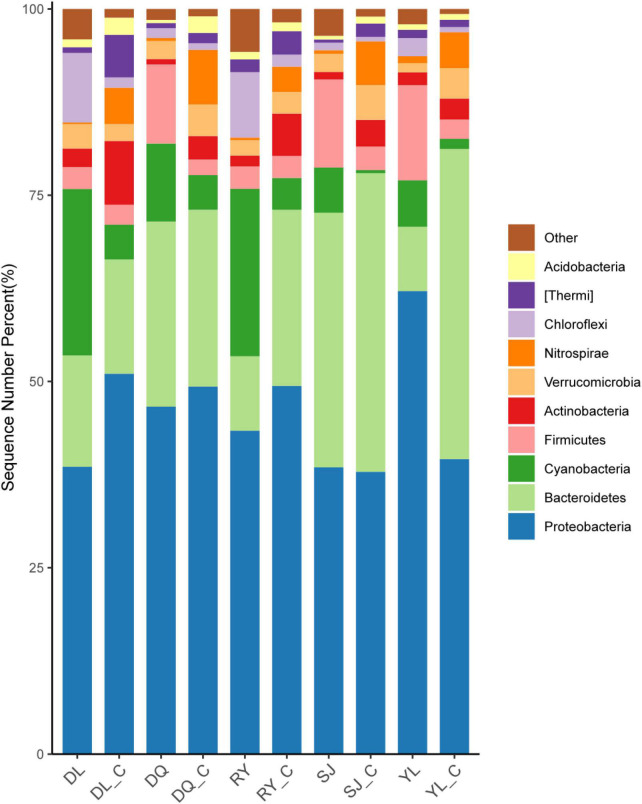
Changes of *in situ* microbial populations of bio-eroded shells in different aquatic environment. DL, DQ, RY, SJ, and YL represent the microbial communities of bio-eroded shells from five different geographical locations, respectively. DL_C, DQ_C, RY_C, SJ_C, and YL_C represent the microbial communities of the healthy snail shells from five different geographical locations, respectively. SL, Sanjiang County; YL, Yongle Town; DL, Dongliang Village; DQ, Dongquan Town; RY, Renyi Village.

**FIGURE 5:**
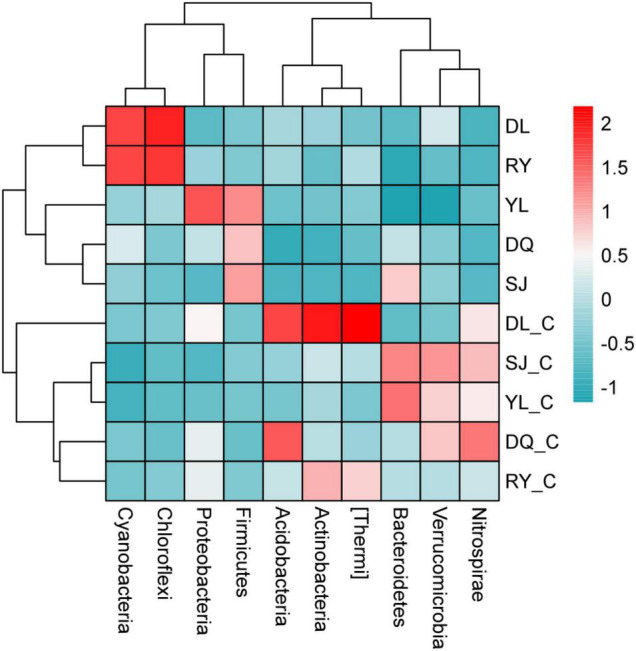
Cluster analysis of microbial populations in different bio-eroded shells. DL, DQ, RY, SJ, and YL represent the microbial communities of bio-eroded shells from five different geographical locations, respectively. DL_C, DQ_C, RY_C, SJ_C, and YL_C represent the microbial communities of the healthy snails shells from five different geographical locations, respectively. SL, Sanjiang County; YL, Yongle Town; DL, Dongliang Village; DQ, Dongquan Town; RY, Renyi Village.

This study analyzed the changes in the microbial community in both bio-eroded and healthy shells in aquatic environment from different geographical locations. The results showed that the top five phyla with the highest abundance were as follows: Proteobacteria, Bacteroidetes, Cyanobacteria, Firmicutes, and Chloroflexi (Figure 4 and Supplementary Table 1). Cluster analysis revealed that the abundance of Cyanobacteria and Chloroflexi was significantly higher compared to other phyla in DL and RY (Figure 5 and Supplementary Table 1). In abundance of Proteobacteria, Firmicutes, and Bacteroidetes was higher in YL, DQ, and SJ, respectively (Figure 5 and Supplementary Table 1). It has been reported that Bacteroidetes and Firmicutes are the dominant beneficial bacteria in the human gut without perforation function (Taib et al., 2020; Pereira et al., 2021), implying that both types of bacteria may not have caused shell bioerosion in this study. While Cyanobacteria have been reported to have bioerosion functions (Prusina et al., 2015), Chloroflexi comprised a large group of linear bacteria with green pigments (Islam et al., 2019). Proteobacteria is the largest phylum of bacteria, including many pathogenic bacteria such as *Escherichia coli*, *Salmonella*, *Vibrio*, and *Helicobacter* (Shin et al., 2015). Therefore, results from this study and previous studies suggested that microorganisms belonging to the three phyla, namely, Cyanobacteria, Chloroflexi, and Proteobacteria, may be responsible for the bioerosion of shells.

### Infectivity of Laboratory-Level Bioerosion

**FIGURE 6:**
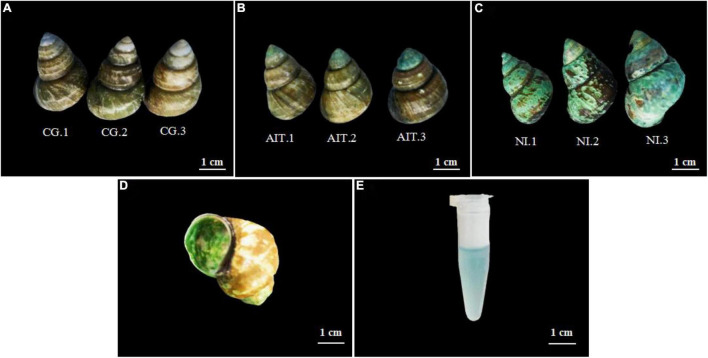
Morphological changes of the snail shell after infection and bioerosion at the laboratory level. **(A)** Healthy snail shell, control group (CG); **(B)** artificial infection group (AIT); **(C)** natural infection bio-eroded experimental group (NI); **(D)** internal morphology of bio-eroded shell; **(E)** phycocyanin infiltrated from the shell.

As *in situ* wild experiments have too many variables to determine the cause of shell erosion, infection experiments were performed at the laboratory level. Results showed no signs of bioerosion in CG shells (Figure 6A). However, the top of the shells in AIT was infected and eroded compared to other parts of the shells (Figure 6B), and the shells from NI were eroded more severely and widely (Figure 6C). These results indicate that the potential bioeroders start infecting shells at the apex (i.e., the oldest part of the shell) and proceed toward the growing shell margin. Indeed, previous studies also reported a similar bioerosion process in the shells (Mao et al., 1996; Prusina et al., 2015). In addition, it was observed that the outer surface of these shells was rough, but the inner surface was smooth (Figure 6D), indicating that these bioeroders horizontally infected the shells, which confirmed the results observed by SEM (Figure 3F). After the blue-like phycocyanin was extracted from the shells (Figure 6E), the shells’ color turned from blue to white. These observations implied that Cyanobacteria may be the leading cause of shell bioerosion by traditional observation methods.

**FIGURE 7:**
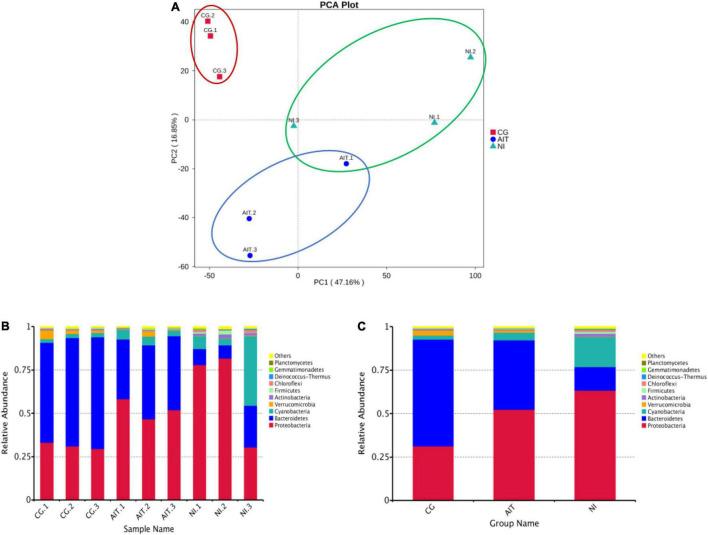
PCA score plot of microbial populations in bio-eroded shells and changes in their relative abundances. **(A)** PCA score plot; **(B)** relative abundance of microbial populations on the shell of a single bio-eroded shells; **(C)** relative abundance of microbial populations on the shell under different treatment groups; (CG) control group. AIT, artificial infection group; NI, natural erosion group.

**FIGURE 8:**
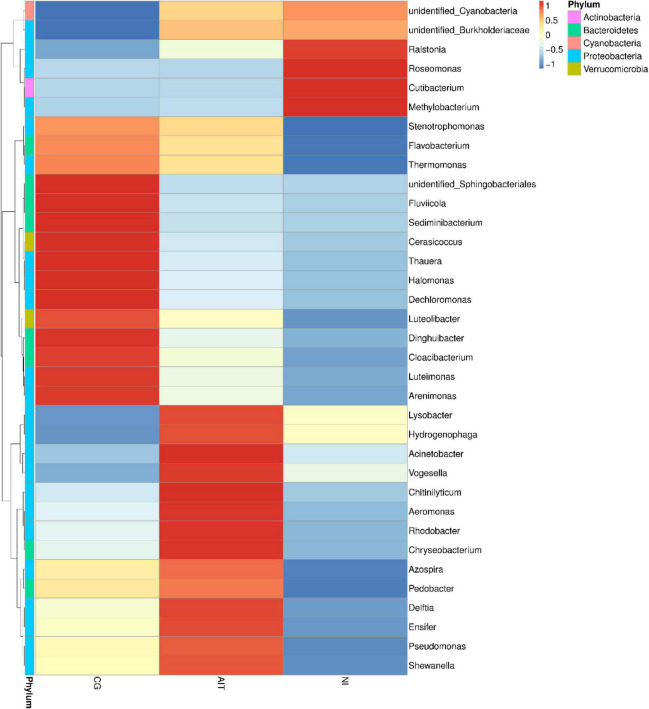
Cluster analysis of different groups of microbial communities at the genus taxonomic level. CG, control group; AIT, artificial infection group, NI, natural erosion group.

To further investigate how the bioeroders infected healthy snail shells and whether there was an overlap of microbial population between AIT and NI, we used the 16S rRNA amplification and sequencing technique. The microbial overlap was clearly distinguished from the CG (Figure 7A), indicating that the potential bioeroders were infectious, and these overlapping microbes were crucial for causing shell bioerosion. Compared with the wild environment, the laboratory conditions were more controllable and three significant microbial populations, namely, Proteobacteria, Cyanobacteria, and Bacteroidetes, were observed. Results showed that Bacteroidetes in CG had the highest relative abundance (0.63), followed by Proteobacteria (0.32) and Cyanobacteria (0.03) (Figure 7C and Supplementary Table 2). In contrast, the relative abundance of Bacteroidetes in AIT was 0.37 (*p* < 0.0001), Proteobacteria was 0.53 (*p* < 0.0001), and Cyanobacteria was 0.08 (*p* < 0.0332). In NI, the proportion of Bacteroidetes was further compressed to 0.13 (*p* < 0.0001), Proteobacteria increased to 0.63 (*p* < 0.0002), and Cyanobacteria increased to 0.21 (*p* < 0.0021). In particular, the more severely bioerosion in the shells, the greater the relative abundance of Cyanobacteria (Figures 6C, 7B and Supplementary Table 2). In addition, in the NI group, Chloroflexi accounted for only 0.01% of abundance Figure 7C. It was almost undetectable in the AIT group, indicating that Chloroflexi were not infectious and may not trigger erosion in healthy snail shells (Xian et al., 2020). It is well known that Bacteroidetes are beneficial bacteria for the snail shells. When the relative abundance of the bacteria got reduced, other harmful microorganisms became dominant, resulting in shell’s bioerosion. Therefore, results from this study provide some evidence that the primary microorganisms responsible for the bioerosion of snail shells were Cyanobacteria and Proteobacteria. Cluster analysis at the genus level revealed the unidentified Cyanobacteria and unidentified Burkholderiaceae and *Raistonia* spp. of the phylum Proteobacteria (Figure 8). The predicted functional analysis of 16S rRNA showed that the functions of organic systems, cellular processes, and environmental information were significantly upregulated in AIT and NI (Supplementary Figure 1), implying that Cyanobacteria and Proteobacteria had frequent metabolic exchanges of substances and may promote bioerosion of snail shells.

### The Interaction Between Cyanobacteria and Proteobacteria Promotes Bioerosion of Shells

**FIGURE 9:**
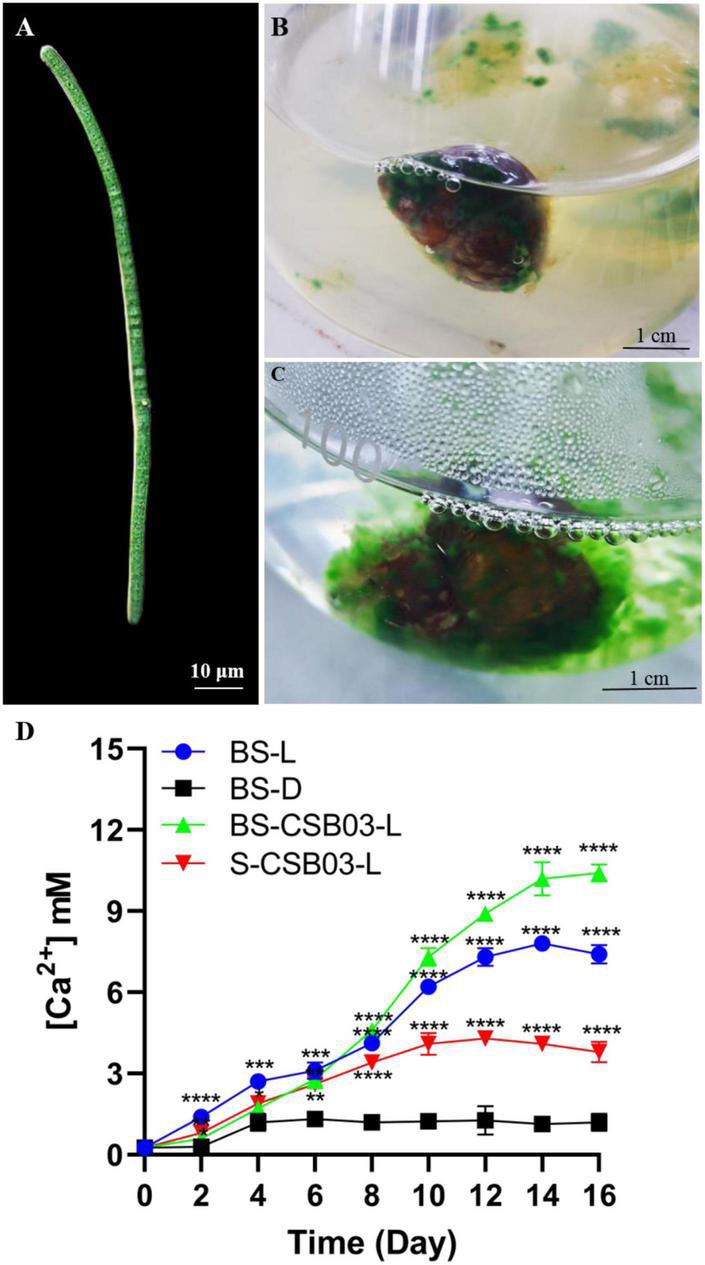
Cyanobacterial CSB03 morphology and changes in Ca^2+^ concentration during the bioerosion of snail shells. **(A)** Morphology of cyanobacterial CSB03; **(B)** the early stage of Cyanobacteria eroding the snail shell; **(C)** the later stage of Cyanobacteria eroding the snail shell; **(D)** the change of Ca^2+^ concentration in the medium during the bioerosion process of the snail shell. BS-D, control group, which no light, 30°C conditions; BS-L, experimental group under 100 μmol m^–2^s^–1^ light; BS-CSB03-L, CSB03 algal cells were inoculated into the medium; S-CSB03-L, the bio-eroded shells were highly sterilized. The values represent mean ± SD (*n* = 3). Analysis of significant differences of control and experiment groups was performed using one-way ANOVA with Sidak’s *post-test* for multiple comparisons. *p < 0.0332; **p < 0.0021; ***p < 0.0002; ****p < 0.0001.

Results showed that the bioerosion of the snail shells may be caused by the interaction between Cyanobacteria and specific bacteria. In this study, a filamentous cyanobacterium was successfully isolated, which can penetrate and grow in solid agar, unlike other green algae (such as *Chlorella*) that only form circular algal colonies on the surface of the solid agar plates (Supplementary Figure 2). Microscope observation revealed the filamentous Cyanobacteria (about 3–5 μm wide), preliminarily identified as *Schizothrix* spp. and named CSB03 (Figure 9A). Such characteristics suggest that the CSB03 may implant in calcium carbonate substances, similarly to the cyanobacterial BC008 with bioerosion function (Garcia-Pichel et al., 2010). When cultivated together, filamentous CSB03 can entangle with the snail shell and gradually wrap around the shell surface (Figures 9B,C), suggesting that Cyanobacteria may be responsible for the bioerosion of snail shell. It is wildly accepted that the changes in concentration of calcium ions in the culture solution indirectly reflect the bioerosion of the shells on indirectly reflecting the bioerosion of the shells (Garcia-Pichel et al., 2010). When the bio-eroded snail shells were incubated in the dark, the Ca^2+^ concentration increased from an initial concentration of 0.25–1.2 mM on day 4, and its concentration was almost unchanged in subsequent cultures (Control, BS-D). The Ca^2+^ concentration of BS-L in the medium was significantly increased under light conditions, especially by day 16, its concentration reached 7.4 mM, which was 6.17 times that of BS-D (Figure 9D, *P* < 0.0001). These results suggest that the bioerosion of snail shell occurred under light conditions. In the presence of the isolated *Cyanobacteria* CSB03, the bioerosion of the snail shell was more obvious. For instance, on the 16th day of culture, the Ca^2+^ concentration of BS-CSB03-L reached 10.4 mM, which was 8.67 times higher than that of the CG (Figure 9D, *P* < 0.0001). Under the sterile condition, the Ca^2+^ concentration of S-CSB03-L was 3.8 mM on day 16, which was 3.17 times higher than that of the BS-D group (Figure 9D, *P* < 0.0001). Comprehensive analysis showed that the bioerosion of snail shells was accelerated with under the interaction of photosynthetic Cyanobacteria and bacteria. These findings highlight that shell bioerosion was not only associated with Cyanobacteria but also with the interactions between Cyanobacteria and bacteria (especially Proteobacteria). Nevertheless, more research is needed to determine the identity of harmful bacteria that interact with Cyanobacteria.

Numerous previous studies reported that shell bioerosion is caused by euendolithic pseudofilamentous and filamentous Cyanobacteria, namely, *Hormathonema paulocellulare*, *Hyella caespitosa*, *Mastigocoleus testarum*, and *Leptolyngbya* sp. (Prusina et al., 2015). During the bioerosion of a shell, Cyanobacteria absorb Ca^2+^ from the shell through their Ca^2+^ channels, secrete the ions outside of the shell, and create concentration differences within the shell, promoting the dissolution of CaCO_3_ (Garcia-Pichel et al., 2010). The SEM revealed the loose calcareous surface layer on the surface of the shell, which is likely due to the calcium precipitation caused by Cyanobacteria transporting the calcium from the shell. This study also found some tiny pores on the surface of the shell likely caused by bacteria (e.g., *Ralstonia* spp.) with flagella in the phylum Proteobacteria. For example, *Ralstonia eutropha* can aggravate oxalates (as a carbon and energy source), producing carbonic acid and ultimately Ca^2+^ that could react with locally sourced calcium to produce calcite (De Boever et al., 2017).

**FIGURE 10:**
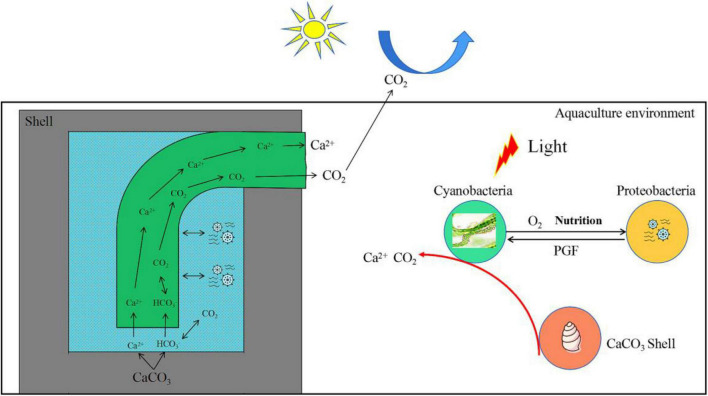
Model diagram of interaction between Cyanobacteria and Proteobacteria to promote the bioerosion of snail shells. PGF, promote growth factors.

Additionally, the green alga, *Ostreobium*, has been reported to supply polysaccharides and essential growth factors such as vitamin B_12_, enabling the degradation of skeletal CaCO_3_ (Iha et al., 2021). Similarly, Cyanobacteria and harmful bacteria in this study were not only nutritionally complementary but also mutually promoted shell bioerosion (Figure 10). In addition, in the wild aquatic environment, it was observed that the later the shell erosion, the faster the degree of bioerosion (data not shown).

## Conclusion

The phenomenon of “shell bioerosion” caused by microorganisms in aquatic environments has been widely reported. These bioeroders could be implanted in the CaCO_3_ layer of the snail shell, resulting in many small holes that reduce the density of a shell and make the shell fragile. Results of the study showed that microorganisms from phyla, namely, Cyanobacteria and Proteobacteria are likely to be responsible for the bioerosion of snail shells. In addition, the interaction of Cyanobacteria and Proteobacteria may have triggered the bioerosion of shells. This study is helpful in elucidating the causes of shell bioerosion in farmed animals across aquatic environments. The study further provides a new theoretical basis and research direction for additional studies that clarify the mechanism by which Cyanobacteria and bacteria interact to trigger the erosion of shells composed of CaCO_3_.

## Data Availability Statement

The data has been deposited to NBCI with accession number PRJNA852667.

## Author Contributions

MW and GW conceived and designed the experiments and analyzed the data. GW, FL, and HW performed the experiments. FL provided research found for the manuscript. AH, YW, and JW revised the manuscript. All authors read and approved the final manuscript.

## Conflict of Interest

The authors declare that the research was conducted in the absence of any commercial or financial relationships that could be construed as a potential conflict of interest.

## Publisher’s Note

All claims expressed in this article are solely those of the authors and do not necessarily represent those of their affiliated organizations, or those of the publisher, the editors and the reviewers. Any product that may be evaluated in this article, or claim that may be made by its manufacturer, is not guaranteed or endorsed by the publisher.
