# Supplementary material for: Euendolithic Cyanobacteria and Proteobacteria Together Contribute to Trigger Bioerosion in Aquatic Environments
Source: Front Microbiol. 2022 Jul 6;13:938359. doi: 10.3389/fmicb.2022.938359 (PMC9298513; doi:10.3389/fmicb.2022.938359)
Supplement: Supplementary file 1 [file Table_1.docx]

| **Taxonomic phylum** | **DL (*p* value)** | | **DQ (*p* value)** | | **RY (*p* value)** | | **SJ (*p* value)** | | **YL (*p* value)** | |
| --- | --- | --- | --- | --- | --- | --- | --- | --- | --- | --- |
| Chloroflexi | <0.0001 | **** | >0.9999 | ns | 0.0244 | * | >0.9999 | ns | >0.9999 | ns |
| Cyanobacteria | <0.0001 | **** | 0.0274 | * | <0.0001 | **** | 0.0201 | * | 0.0287 | * |
| Verrucomicrobia | 0.9988 | ns | >0.9999 | ns | >0.9999 | ns | 0.9979 | ns | 0.9998 | ns |
| Firmicutes | >0.9999 | ns | 0.2418 | ns | >0.9999 | ns | 0.0590 | ns | 0.4307 | ns |
| Bacteroidetes | >0.9999 | ns | >0.9999 | ns | 0.0001 | *** | 0.0234 | * | <0.0001 | **** |
| Proteobacteria | <0.0001 | **** | 0.0298 | * | 0.0305 | * | >0.9999 | ns | 0.0008 | *** |
| Acidobacteria | 0.9928 | ns | >0.9999 | ns | >0.9999 | ns | >0.9999 | ns | >0.9999 | ns |
| Actinobacteria | 0.0014 | ** | 0.9993 | ns | 0.7601 | ns | 0.9934 | ns | >0.9999 | ns |
| [Thermi] | 0.0143 | * | >0.9999 | ns | >0.9999 | ns | >0.9999 | ns | >0.9999 | ns |
| Nitrospirae | 0.0241 | * | 0.5112 | ns | 0.9612 | ns | 0.5613 | ns | 0.9975 | ns |

**Supplementary Table 1** Analysis of significant differences in relative abundance of the top 10 taxonomic phylum of the bio-eroded shells in different geographical locations

DL, DQ, RY, SJ, and YL represented the relative abundance of microbial communities of the bio-eroded shells from 5 different geographical locations, respectively; SL, Sanjiang County; YL, Yongle town; DL, Dongliang Village; DQ, Dongquan town; RY, Renyi Village. Analysis of significant differences of control and experiment groups were performed using 2-way ANOVA with Sidak’s post-test for multiple comparisons, ns, *p* value > 0.1234 ; * , *p* value < 0.0332; **, *p* value < 0.0021; ***, *p* value <0.0002; ****, *p* value < 0.0001.

**Supplementary Table 2** Analysis of significant differences in relative abundance of the top 10 taxonomic phylum of the bio-eroded shells at the laboratory level

| **Taxonomic phylum** | **AIT & CG (*p* value)** | | **NI & CG (*p* value)** | | **AIT & NI (*p* value)** | |
| --- | --- | --- | --- | --- | --- | --- |
| Proteobacteria | <0.0001 | **** | 0.0002 | *** | 0.0191 | * |
| Bacteroidetes | <0.0001 | **** | <0.0001 | **** | 0.0016 | ** |
| Cyanobacteria | 0.0251 | * | 0.0016 | ** | 0.0206 | * |
| Verrucomicrobia | 0.9645 | ns | >0.9999 | ns | >0.9999 | ns |
| Actinobacteria | >0.9999 | ns | >0.9999 | ns | >0.9999 | ns |
| Firmicutes | >0.9999 | ns | >0.9999 | ns | >0.9999 | ns |
| Chloroflexi | >0.9999 | ns | >0.9999 | ns | >0.9999 | ns |
| Deinococcus-Thermus | >0.9999 | ns | >0.9999 | ns | >0.9999 | ns |
| Gemmatimonadetes | >0.9999 | ns | >0.9999 | ns | >0.9999 | ns |
| Planctomycetes | >0.9999 | ns | >0.9999 | ns | >0.9999 | ns |

CG, control group; AIT, artificial infection group; NI, natural infection bio-eroded experimental group. Analysis of significant differences were performed using 2-way ANOVA with Sidak’s post-test for multiple comparisons. ns, *p* value > 0.1234; *, *p* value < 0.0332; **, *p* value < 0.0021; ***, *p* value < 0.0002; ****, *p* value < 0.0001.


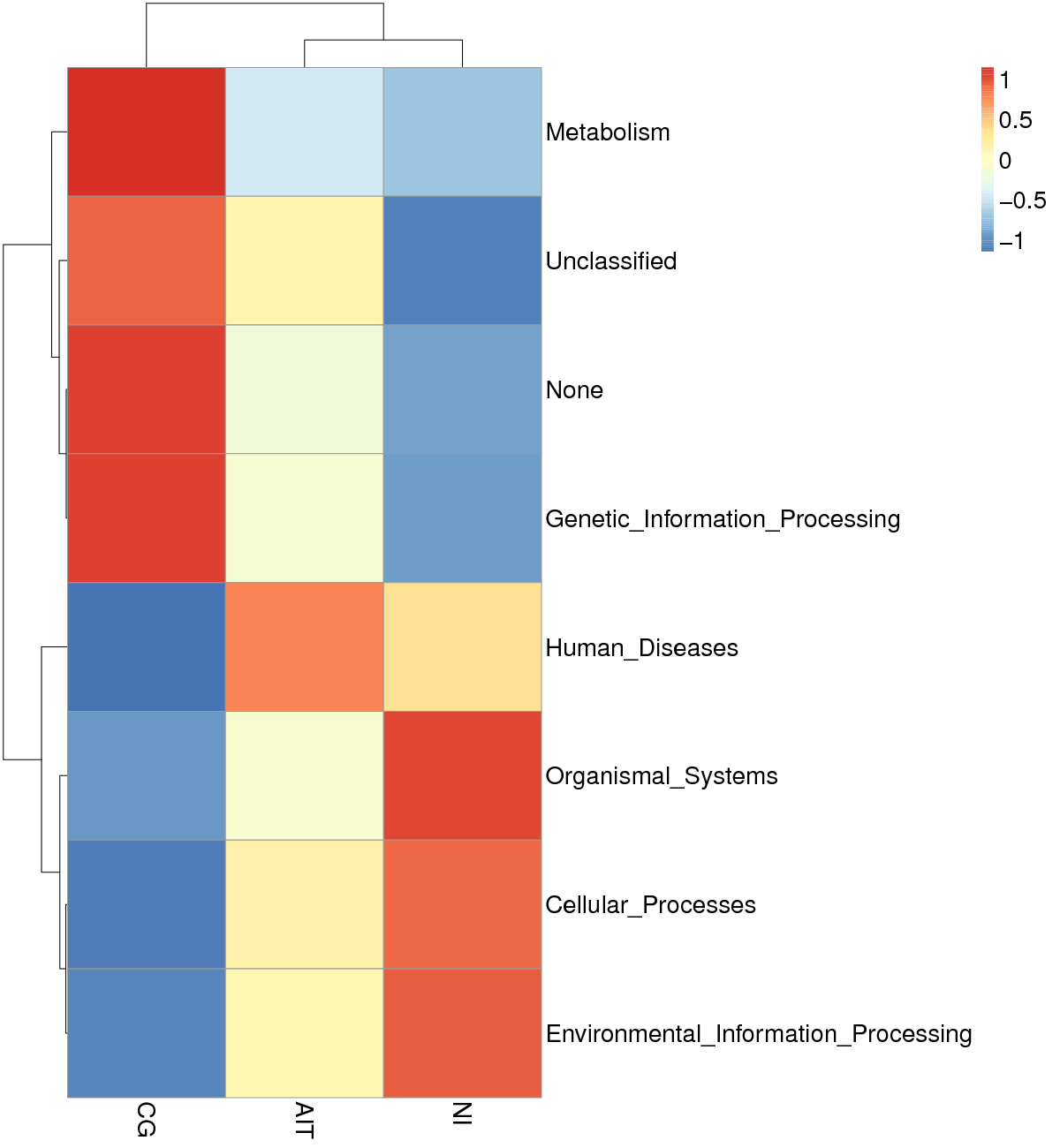


**Supplementary Figure 1 Cluster analysis for predicting the function of microorganisms at the phylum level.** CG, control group; AIT, artificial infection test group; NI, natural infection bio-eroded group.


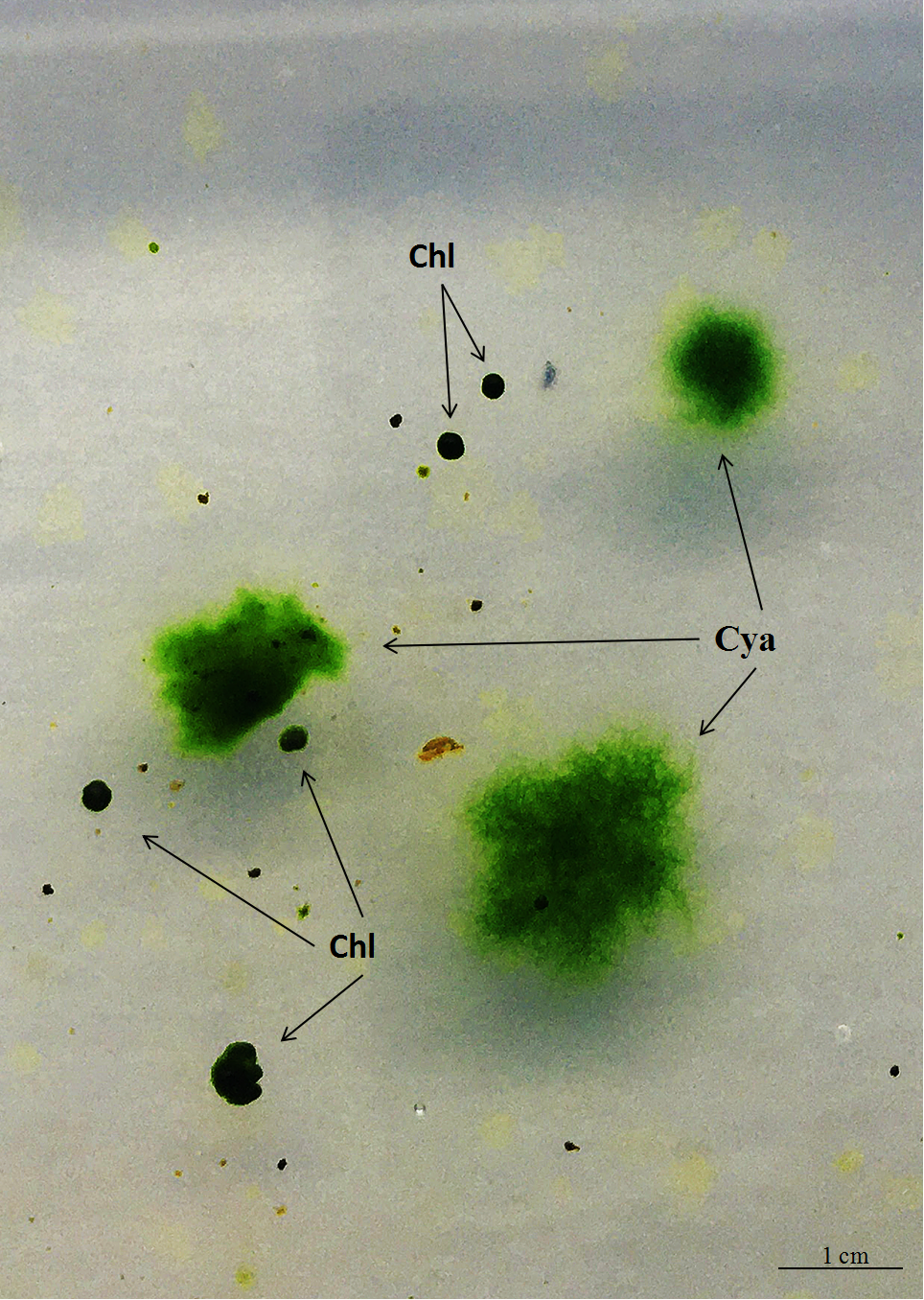


**Supplementary Figure 2 Colony morphology of screened microalgae growing on solid medium.** Chl, Chlorella; Cya, Cyanobacteria.
